# Alterations in the autonomic and haemodynamic response to prolonged high‐intensity endurance exercise in individuals with coronary artery calcification

**DOI:** 10.1113/EP092201

**Published:** 2024-12-31

**Authors:** Jakob Svane, Tomasz Wiktorski, Trygve Eftestøl, Stein Ørn

**Affiliations:** ^1^ Department of Electrical Engineering and Computer Science University of Stavanger Stavanger Norway; ^2^ Division of Cardiology Stavanger University Hospital Stavanger Norway

**Keywords:** coronary artery calcification, exercise, heart rate variability

## Abstract

Endurance exercise is associated with increased life duration and improved life quality. Paradoxically, high exercise intensity is also associated with increased coronary artery calcification (CAC) and a small but significant increased risk of adverse cardiac events during exercise. The mechanisms underlying the development of CAC during prolonged high‐intensity endurance exercise are unknown. This study aims to determine if there are differences in cardiovascular haemodynamic measures and heart rate variability (HRV) in individuals with (CAC^+^) and without CAC (CAC^−^). Hemodynamic measures from 56 healthy, middle‐aged (median [interquartile range] 51 [43–58] years) individuals (41 men/15 women) participating in a 91 km [251.2 [217.2‐271.6] min] leisure sport mountain bike race were included in this study. Twenty‐five participants (20 men/5 women) were classified as CAC^+^ based on coronary computed tomographic assessment. Haemodynamic measures and HRV were quantified at the top of the hardest hill (THH) during the last quarter of the race. At the top of THH, CAC^+^ individuals had significantly higher systolic blood pressure (SBP) (235 [225–245] mmHg vs. 220 [193–238] mmHg, *P* = 0.008), higher diastolic blood pressure (DBP) (105 [95–110] mmHg vs. 95 [85–110] mmHg, *P* = 0.006), higher pulse pressure (130 [125–140] mmHg vs. 123 [110–130] mmHg, *P* = 0.039), higher mean rate pressure product (33,882 [30,872–35,053] bpm × mmHg vs. 31,028 [27,392–33,047] bpm × mmHg, *P* = 0.028), and larger increase in DBP from baseline (20 [20–30] mmHg vs. 10 [0–20] mmHg, *P* = 0.001), compared with CAC^−^ individuals. Further, CAC^+^ participants showed a significant reduction in the low‐frequency component of HRV (HRV_LF_) (6.3 [2.4–11.5] ms^2^ vs. 12.4 [6.8–20.2] ms^2^, *P* = 0.044). In multivariable analysis, HRV_LF_ was an independent predictor of the presence of CAC even after adjusting for established risk factors of atherosclerosis: age, sex, body mass index, maximum heart rate, ${\dot{V}}_{O_{2}\max}$, smoking, resting SBP and resting DBP. CAC^+^ individuals had significant alterations in haemodynamic measures and HRV_LF_ following prolonged high‐intensity endurance exercise compared with individuals without CAC. HRV_LF_ was an independent predictor of CAC, suggesting an adverse autonomic response to high‐intensity endurance exercise in individuals with CAC.

## Abstract (highlights)

- **What is the central question of this study?**
  Coronary artery calcification (CAC) is a marker of coronary artery disease: how do individuals with CAC respond in terms of haemodynamic and autonomic measures to prolonged high‐intensity endurance exercise compared to those without CAC?
- **What is the main finding and its importance?**
  Individuals with CAC have higher blood pressure and a lower low‐frequency component of heart rate (HR) variability in response to prolonged high‐intensity exercise compared with individuals without CAC. These findings indicate that blood pressure and HR variability measures may be potential monitoring tools for individuals with CAC.

## 1 INTRODUCTION

Endurance exercise is associated with a decreased risk of cardiovascular disease (CVD) and reduced progression of coronary artery disease (CAD) (Eijsvogels et al., 2016; Shiroma & Lee, 2010). However, there is an increased risk of adverse cardiac events during exercise (Thompson et al., 2007). Recent studies have demonstrated an increased risk of coronary artery calcification (CAC) in individuals exposed to prolonged high‐intensity endurance exercise (Aengevaeren et al., 2023; Hou et al., 2012; Sangiorgi et al., 1998; Silverman et al., 2014). The mechanisms of the association between prolonged high‐intensity endurance exercise and increased CAC are unknown.

The present study aims to determine if there are differences in the physiological response to prolonged high‐intensity endurance exercise in individuals with and without CAC, comparing individuals without CAC (CAC^−^) to individuals with CAC without coronary artery obstruction (CAC^+^). The main physiological parameters assessed in this study were blood pressure, heart rate (HR) and various heart rate variability (HRV) parameters. These parameters were selected due to their relevance in evaluating cardiovascular stress and autonomic regulation during and after high‐intensity endurance exercise, as previous research has shown that CVD is associated with impaired cardiovascular autonomic function (Zambach et al., 2023). We hypothesize that the physiological demands of prolonged high‐intensity endurance exercise are associated with different haemodynamic and autonomic responses in CAC^+^ individuals compared with CAC^−^ individuals.

## 2 METHODS

This study analysed a subset of participants from the NEEDED research programme (clinical trial registration https://www.clinicaltrials.gov/; unique identifier: NCT02166216). The NEEDED research programme aims to identify markers and mechanisms of pathological cardiac response to high‐intensity endurance exercise in presumably healthy individuals. The present study (NEEDED 2018) is a mechanistic study of 56 individuals without obstructive CAD assessed by repeated coronary computed tomography angiography (CCTA). The study participants were recruited from previous participants assessed by CCTA in the NEEDED 2013 and 2014 studies (Kleiven et al., 2019; Skadberg et al., 2018). The study individuals repeated participation in the 91 km leisure sport mountain bike race (the North Sea Race) in 2018 (Kleiven et al., 2020).

**FIGURE 1:**
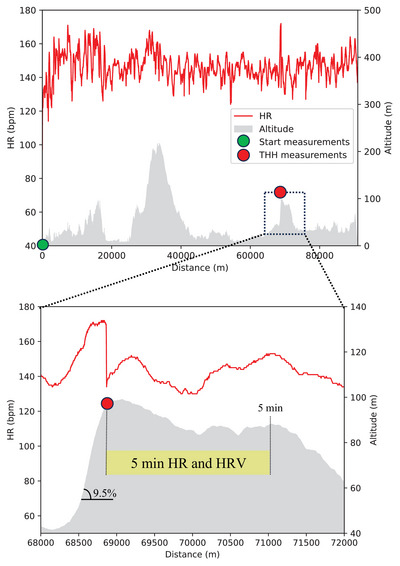
The figure shows the altitude profile of the race and the hardest hill (THH), a hill occurring after 68.5 km into the race, with the red dot marking where the participants temporarily stopped. The red line is the heart rate of one of the participants in the study. The yellow shade represents the approximate distance the participants cycled during the 5‐min heart rate (HR) and heart rate variability (HRV) measurements after the stop.

The present analysis focused on the measurements made at the top of the race's most challenging hill (Tinghaug hill (THH)). THH is in the last quarter of the race (after 68.5 km, 76%) and is 650 m long with an average gradient of 9.5% and a maximum gradient of 16.0%. The course flattens out at the top of the hill, before a slight downhill section of approximately 3.1 km. Figure 1 shows the altitude profile of both the entire race and THH, and where the participants were stopped to measure the haemodynamic markers. The figure includes the HR curve for one of the participants. Blood pressure and lactate were measured before the start and during the race at the top of THH. Wearable sensors measured HR, HRV and power output continuously throughout the race.

### 2.1 Study subjects

At inclusion for NEEDED 2018, a comprehensive medical examination was carried out on all participants, including a detailed medical history, blood tests, electrocardiography, blood pressure measurements and echocardiographic assessment. Baseline characteristics of the subjects, including ${\dot{V}}_{O_{2}\max}$ and maximum and minimum HR measurements, were measured and registered in a laboratory setting 2–3 weeks before the race. CCTA was used to determine the presence of CAC during the week after the race. Subjects with *>*0 Agatston units were classified as CAC^+^, and subjects without CAC were classified as CAC^−^. This study adheres to the *Declaration of Helsinki*, with all participants providing informed consent prior to participation. The study was approved by the regional ethics committee (Regional Etisk Komité (REK) no. 2013/550 and 2018/63).

### 2.2 Data collection

Physiological measurements were collected just before the start of the race, and at the top of THH. The participants stopped briefly to measure the physiological parameters at the top of THH. Blood pressure was measured manually on the right arm with a Heine G5, G7 or XXL LF‐T (Heine, Herrsching, Germany), and the lactate was measured on a finger on the left hand with a Lactatescout Plus (EKF diagnostics, Cardiff, UK). Three lactate measurements were made, with the mean of the two closest values used in the statistical analysis. Time, distance, and altitude were measured using smart watches (Garmin Forerunner 935; Garmin, Olathe, KS, USA), with HR and HRV continuously measured using a Garmin HRM chest strap. Of the 56 participants analysed in this paper, 38 were equipped with Stages power meters (StagesPower, Boulder, CO, USA) on their bikes.

### 2.3 HR data

Continuous HR monitoring was used to assess the max HR reached on THH, the HR recovery during the 30 s immediately after stopping, the mean HR during the stop and the mean HR during the 5 min following the stop.

All HR measurements are reported as absolute values, as fractions of the maximum HR achieved in the ${\dot{V}}_{O_{2}\max}$ test and as the HR reserve,

$$\;\mathrm{HR}\;\mathrm{reserve}=\frac{\mathrm{HR}-HR_{\mathrm{rest}}}{HR_{\max,\mathrm{lab}}-HR_{\mathrm{rest}}}.$$

where HR_max,lab_ is the maximum HR achieved in the ${\dot{V}}_{O_{2}\max}$ test, and HR_rest_ is the participant's self‐reported resting HR.

### 2.4 HRV data

HRV is the variation in time difference between successive heartbeats and is often characterized by statistical measures in both the time domain and the frequency domain (Malik, 1996). We assessed the root mean square of successive differences (HRV_RMSSD_) and the standard deviation of normal–normal intervals (HRV_SDNN_) in the time domain. In the frequency domain, we assessed the very low‐frequency band (HRV_VLF_), 0–0.04 Hz; the low‐frequency band (HRV_LF_), 0.04–0.15 Hz; the high‐frequency band (HRV_HF_), 0.15–0.4 Hz; and the total power (HRV_TP_) of these three bands. The frequency domain metrics are calculated using the fast Fourier transform on an even resampling of the HRV signal. Several time and frequency domain metrics allow for a more comprehensive assessment of sympathetic, parasympathetic, baroreflex and cardiac function (Malik, 1996; Rahman et al., 2011; Reyes del Paso et al., 2013).

HRV metrics were analysed during the 5 min following (and including) the stop. Due to the short stop at THH, true cyclicity in frequency domain metrics may not be apparent during the stop. Baek et al. (2015) found that in ultra‐short‐term HRV measurements, the HRV_LF_ component would need approximately 90 s of measurement to be reliable. For short‐term HRV measurements, recordings of at least 5 min are recommended (Malik, 1996), supporting the legitimacy of the 5 min measurement results after THH.

#### 2.4.1 Preprocessing

The methods used to detect and correct data artifacts can significantly affect the values of the calculated HRV metrics (Cajal et al., 2022; Rincon Soler et al., 2018). Median filters are commonly used for artifact detection. For our data, we found that a median filter with a 5% threshold seemed to yield the best results. We applied deletion, linear interpolation and autoregressive integrated moving average (ARIMA) for artifact correction, based on results from Svane et al. (2023). Cubic interpolation was also applied, since it is the most used method (Giles & Draper, 2018). The HRV metrics were then calculated based on the corrected signal from each method. Also the raw signal was analysed.

### 2.5 Rate pressure product

Rate pressure product (RPP) was used to estimate cardiac work. RPP is defined as the product of systolic blood pressure (SBP) and HR. Both mean and maximum RPP were calculated, with maximum RPP calculated as SBP multiplied by maximum HR achieved uphill, and mean RPP being SBP multiplied by the mean HR during the stop.

### 2.6 Statistical analysis

All variables were analysed by non‐parametric methods and reported as median and the interquartile range (25th percentile–75th percentile). By applying non‐parametric methods, we ensure robustness and minimize the impact of outliers and potential measurement errors as well as data preprocessing issues. The differences between the two groups were assessed using the non‐parametric Mann–Whitney test, with categorical variables assessed using the chi‐square test. A two‐tailed *P*‐value *<*0.05 was considered statistically significant. A multivariable logistic regression with CAC as the dependent variable was used to assess the effect of the variables with significant differences between CAC^+^ and CAC^−^. The SciPy library (Virtanen et al., 2020) in Python (Python Software Foundation, 2020) was used for the statistical analysis.

## 3 RESULTS

Baseline data, comparing individuals with and without CAC, are presented in Table 1. The median race duration was 251.2 (217.2–271.6) min. The participants spent 268 (239–311) s going uphill at THH, and 129 (101–178) s at the top of the hill during the data‐collection stop. There were no differences between groups for finish time, uphill duration or duration of stop for data collection. Among the participants equipped with power meters, the absence of power output indicated that six participants stepped off the bike and walked a portion while ascending the hill. Inspection of the speed up the hill showed that another six participants without power meters also stepped off the bike. Thus, 12 of 56 participants (six CAC^−^ and six CAC^+^) stepped off the bike during the uphill climb. The participants who stepped off the bike had a significantly lower ${\dot{V}}_{O_{2}\max}$ compared to those who did not (*P* = 0.003).

**TABLE 1:** Baseline characteristics of the two groups.

	CAC^−^	CAC^+^	*P*
Number of subjects	31	25	N.A.
Male/female sex	21/10	20/5	0.468
Age (years)	46 (41–52)	55 (50–59)	0.001
Body mass index (kg/m^2^)	24.9 (23.3–26.1)	25.1 (23.9–27.6)	0.277
Systolic blood pressure (mmHg)	135 (130–150)	145 (130–150)	0.170
Diastolic blood pressure (mmHg)	80 (80–90)	80 (75–85)	0.464
Pulse pressure (mmHg)	50 (47–65)	60 (50–75)	0.149
Resting HR (beats/min)	54 (49–60)	48 (45–61)	0.349
Lactate (mmol/L)	1.7 (1.3–2.2)	1.7 (1.4–2.1)	0.888
CAC (Agatston units)	0.0	21.0 (5.6–83.0)	N.A.
Maximal HR (beats/min)	180 (173–187)	172 (168–181)	0.163
${\dot{V}}_{O_{2}\max}$ (mL/kg/min)	43.4 (38.0–49.4)	38.3 (34.5–44.9)	0.053

*Note*: Data are presented as median (interquartile range). *P*‐value is given by the Mann–Whitney test.

Abbreviations: CAC, coronary artery calcification; CAC^+^ and CAC^−^, with and without CAC, respectively; HR, heart rate; N.A., not applicable.

### 3.1 Physiological markers

Table 2 shows the median values, *U*‐statistics and *P*‐values at the start of the race and at THH. No statistically significant differences existed between the CAC^−^ and CAC^+^ groups in HR data, whether calculated as absolute values, relative to max, HR reserve or HR recovery. In contrast, blood pressure measurements demonstrated that CAC^+^ had significantly higher SBP (*P* = 0.008), diastolic blood pressure (DBP) (*P* = 0.006) and pulse pressure (PP) (*P* = 0.039) at the top of the hill compared to CAC^−^. In addition, CAC^+^ had a significantly larger increase in DBP from baseline to the top of THH compared with CAC^−^ (*P* = 0.001), and a higher mean RPP than CAC^−^ (*P* = 0.028). Of HRV metrics, CAC^+^ had significantly lower HRV_LF_ measured during the 5 min following the stop (*P* = 0.044).

**TABLE 2:** Comparison of measurements made at the hardest hill and directly before start for the two groups.

	CAC^−^	CAC^+^	*U*	*P*‐value
Number of subjects	31	25		
Finish time (min)	241.6 (215.4–267.8)	255.3 (227.3–273.3)	299	0.147
Time uphill (s)	260 (239–297)	290 (247–320)	315	0.232
Time stop (s)	129 (101–179)	120 (110–179)	383	0.928
Relative power uphill (W/kg)	2.9 (2.4–3.1)	2.8 (2.6–3.0)	179	0.988
Heart rate stats				
HR max uphill (bpm)	171 (165–179)	170 (160–174)	434	0.448
Max HR % uphill	94.7 (92.7–97.9)	95.4 (93.9–99.4)	328	0.432
Max HR reserve uphill	92.1 (88.9–97.0)	93.8 (90.3–102.3)	129	0.494
HR recovery stop (bpm)	12 (10–15)	11 (8–14)	439	0.258
Mean HR stop (bpm)	141 (134–152)	142 (138–148)	369	0.767
Mean HR % stop	79.1 (72.8–81.5)	83.0 (76.0–84.3)	281	0.114
Mean HR reserve stop	69.3 (62.9–71.8)	73.2 (69.3–80.3)	95	0.069
Mean HR 5 min (bpm)	142 (136–152)	142 (137–148)	412	0.692
Mean HR % 5 min	79.4 (74.8–83.6)	81.1 (77.1–84.0)	325	0.403
Mean HR reserve 5 min	70.6 (63.8–73.3)	72.2 (66.9–78.7)	120	0.325
Blood pressure				
SBP start (mmHg)	135 (130–150)	145 (130–150)	305	0.170
** SBP THH (mmHg)**	**220 (193–238)**	**235 (225–245)**	**228**	**0.008**
∆SBP (mmHg)	75 (58–103)	95 (80–105)	275	0.063
DBP start (mmHg)	80 (80–90)	80 (75–85)	432	0.464
** DBP THH (mmHg)**	**95 (85–110)**	**105 (95–110)**	**223**	**0.006**
** ∆DBP (mmHg)**	**10 (0–20)**	**20 (20–30)**	**185**	**0.001**
PP start (mmHg)	50 (48–65)	60 (50–75)	300	0.149
** PP THH (mmHg)**	**123 (110–130)**	**130 (125–140)**	**263**	**0.039**
∆PP (mmHg)	65 (50–80)	70 (55–85)	356	0.602
Max RPP (bpm × mmHg)	37,200 (32763–40717)	40,800 (36,800–42,550)	273	0.059
** Mean RPP (bpm × mmHg)**	**31,028 (27,392–33,047)**	**33,882 (30,872–35,053)**	**254**	**0.028**
Lactate THH	4.3 (3.4–5.8)	5.0 (3.9–6.2)	313	0.219
HRV 5 min				
RMSSD (ms)	4.4 (3.5–5.4)	4.3 (3.6–5.5)	402	0.818
SDNN (ms)	27.9 (22.8–33.3)	25.4 (18.3–30.2)	440	0.391
VLF (ms^2^)	92.6 (58.9–134.4)	64.9 (43.1–94.3)	478	0.138
** LF (ms^2^)**	**12.4 (6.8–20.2)**	**6.3 (2.4–11.5)**	**510**	**0.044**
HF (ms^2^)	2.3 (1.0–6.4)	1.6 (0.9–3.1)	472	0.166
Total power (ms^2^)	110.1 (80.7–164.8)	70.5 (50.8–94.7)	496	0.075

*Note*: Variables with significant differences are highlighted in bold. Reported as median (interquartile range), as well as *U*‐statistic and *P*‐value from Mann–Whitney test. Abbreviations: ∆, change from start to top of the hill; CAC, coronary artery calcification; CAC^+^ and CAC^−^, with and without CAC, respectively; DBP, diastolic blood pressure; HF, high frequency; HR, heart rate; HRV, heart rate variability; LF, low frequency; PP, pulse pressure; RPP, rate pressure product; RMSSD, root mean square of successive differences; SBP, systolic blood pressure; SDNN, standard deviation of normal‐normal interval; THH, the hardest hill; VLF, very low frequency.

The significance of the parameter differences was more pronounced in the participants who cycled up the entire hill than in those who stopped cycling and walked the remainder of the hill. Among those who cycled all the way up THH, individuals with CAC^+^ had significantly higher maximum RPP. These results are displayed in Table 3.

**TABLE 3:** Comparison of measurements made at the hardest hill and directly before start for all participants who cycled the entire hill.

	CAC^−^	CAC^+^	*U*	*P*
Number of subjects	25	19		
Finish time (min)	238.7 (216.7–263.2)	248.1 (220.2–265.4)	209	0.507
Time uphill (s)	258 (239–277)	265 (237–291)	209	0.507
Time stop (s)	124 (101–156)	133 (111–182)	221	0.696
Relative power uphill (W/kg)	2.9 (2.7–3.1)	3.0 (2.6–3.1)	126	0.734
Heart rate stats				
HR max uphill (bpm)	172 (165–182)	172 (168–177)	248	0.822
Max HR % uphill	95.1 (92.9–98.0)	96.3 (94.3–101.6)	193	0.399
Max HR reserve uphill	92.1 (88.9–97.6)	94.8 (92.2–103.5)	63	0.227
HR recovery stop (bpm)	12 (10–15)	11 (8–14)	278	0.195
Mean HR stop (bpm)	141 (135–155)	143 (139–148)	217	0.636
Mean HR % stop	79.2 (75.7–82.3)	83.1 (77.1–84.9)	178	0.226
Mean HR reserve stop	70.7 (62.9–71.8)	79.9 (69.3–82.8)	52	0.080
Mean HR 5 min (bpm)	143 (136–152)	143 (138–148)	238	1.000
Mean HR % 5 min	79.9 (76.3–84.3)	82.5 (77.2–85.3)	196	0.441
Mean HR reserve 5 min	70.6 (63.8–72.6)	74.8 (69.2–80.4)	57	0.132
Blood pressure				
SBP start (mmHg)	130 (125–150)	145 (135–153)	165	0.083
**SBP THH (mmHg)**	**220 (190–235)**	**240 (230–250)**	**110**	**0.002**
∆SBP (mmHg)	75 (60–105)	95 (80–105)	156	0.055
DBP start (mmHg)	80 (80–90)	80 (75–88)	270	0.447
**DBP THH (mmHg)**	**95 (80–110)**	**105 (103–110)**	**135**	**0.014**
**∆DBP** (**mmHg)**	**10 (0–20)**	**20 (20–30)**	**112**	**0.003**
PP start (mmHg)	50 (45–60)	60 (50–75)	161	0.070
**PP THH (mmHg)**	**120 (110–130)**	**130 (125–143)**	**111**	**0.002**
∆PP (mmHg)	65 (50–80)	70 (63–85)	204	0.432
**Max RPP (bpm × mmHg)**	**37,125 (33,350–41,160)**	**41,400 (39,160–42,735)**	**122**	**0.006**
**Mean RPP (bpm × mmHg)**	**30,453 (28,082–33,177)**	**34,706 (32,202–35,630)**	**122**	**0.006**
Lactate THH	4.6 (3.6–5.7)	5.5 (4.0–6.6)	189	0.250
HRV 5 min				
RMSSD (ms)	4.6 (3.8–6.2)	4.3 (3.5–5.3)	268	0.477
SDNN (ms)	27.9 (22.8–33.3)	25.4 (18.4–30.7)	270	0.448
VLF (ms^2^)	79.4 (57.5–124.5)	54.4 (39.7–86.6)	298	0.155
**LF (ms^2^)**	**13.1 (7.1–20.1)**	**5.0 (2.0–8.3)**	**337**	**0.019**
HF (ms^2^)	2.3 (1.0–6.6)	1.4 (0.9–2.8)	309	0.093
Total power (ms^2^)	90.9 (84.3–175.5)	58.0 (44.8–92.5)	309	0.093

Variables with significant differences are highlighted in bold. Reported as median (interquartile range), as well as *U*‐statistic and *P*‐value from Mann–Whitney test. CAC, coronary artery calcification; ∆, change from start to top of the hill; CAC^+^ and CAC^−^, with and without CAC, respectively; DBP, diastolic blood pressure; HF, high frequency; HR, heart rate; HRV, heart rate variability; LF, low frequency; PP, pulse pressure; RPP, rate pressure product; RMSSD, root mean square of successive differences; SBP, systolic blood pressure; SDNN, standard deviation of normal‐normal interval; THH, the hardest hill; VLF, very low frequency.

Table 4 shows the results of the multivariable logistic regression. An increase in the 5 min low‐frequency component of HRV (HRV_LF5min_) was independently associated with a lower risk of having calcified plaques (odds ratio (OR) 0.89, 95% confidence interval (CI) 0.81–0.98, *P* = 0.021), after adjusting for the other significant variables in Table 2. Increased change in DBP from baseline was also associated with an independently increased risk of CAC^+^ (OR 1.12, 95% CI 1.04–1.21, *P* = 0.002).

**TABLE 4:** Odds ratio (OR) from multivariate logistic regression for the presence of calcified plaques, including all participants (*n* = 56).

	OR (95% CI)	*P*
HRV_LF5min_	0.89 (0.81–0.98)	0.021
SBP THH	1.02 (0.96–1.08)	0.596
DBP THH	1.01 (0.92–1.11)	0.870
∆DBP	1.12 (1.04–1.21)	0.002
∆PP	0.97 (0.93–1.01)	0.138
Mean RPP	1.00 (1.0–1.0)	0.423

Abbreviations: ∆, change from baseline to top of the hardest hill; CI, confidence interval; DBP, diastolic blood pressure; HRV_LF5min_, low‐frequency component of heart rate variability, measured 5 min after stopping on top of the hardest hill; PP, pulse pressure; RPP, rate pressure product; SBP, systolic blood pressure; THH, the hardest hill.

### 3.2 HRV

Several methods for artifact correction were applied, with the 5 min HRV_LF5min_ component being significantly different between CAC^+^ and CAC^−^ when applying no correction, deletion, linear interpolation and ARIMA as correction methods. With cubic interpolation, significant differences in HRV_LF5min_ between the two groups were present if the participants who stepped off the bike were removed from the analysis. The HRV data reported in Table 2 were corrected with ARIMA and a 5% threshold.

HRV is influenced by various factors, such as age, sex, and body mass index (Sammito & Böckelmann, 2016). An additional multivariable logistic regression was therefore fitted to ensure that the significance of HRV_LF5min_ is not affected by correlations in HRV and other established risk factors for atherosclerosis. Here, presence of calcified plaques is the dependent variable, with HRV_LF5min_, age, sex, body mass index, maximum HR, ${\dot{V}}_{O_{2}\max}$, smoking, resting SBP, resting DBP and the increase in DBP as the independent variables. In this expanded adjusted model, HRV_LF5min_ remained a significant independent predictor of CAC^+^ (OR 0.80, 95% CI 0.66–0.97, *P* = 0.023), as did the increase in DBP (OR 1.32, 95% CI 1.07–1.62, *P* = 0.009).

## 4 DISCUSSION

The present study demonstrates that non‐obstructive coronary atherosclerosis is associated with a different autonomic and haemodynamic response to prolonged high‐intensity exercise compared to normal coronary arteries. The findings of increased blood pressure in the CAC^+^ group align with previous studies that show exaggerated blood pressure response to exercise in individuals with subclinical vascular impairment compared with healthy normotensive individuals (Miyai et al., 2021). Jae et al. (2006) observed a strong association between carotid atherosclerosis and SBP exceeding 210 mmHg during exercise in healthy men, independent of other established risk factors. This aligns with our findings, where all participants in the CAC^+^ group that completed THH uphill cycling generated SBP that exceeded 210 mmHg.

Vascular resistance and artery stiffness both influence arterial pressure. Vascular resistance increases SBP and DBP, whereas arterial stiffness increases SBP but decreases DBP (Lakatta & Levy, 2003). During exercise, the increase in blood flow and blood pressure can augment these effects, causing significant differences in SBP and PP between the two groups. As perfusion to the coronary arteries happens during diastole, calcification might also affect the perfusion dynamics during diastole. It has been suggested that physical exercise can cause acute smooth muscle relaxation and consequently increased arterial compliance (Saz‐Lara et al., 2021), enhancing the relative effect of arterial resistance compared to arterial stiffness in the CAC^+^ group. This might explain the larger increase in DBP within the CAC^+^ group.

The CAC^+^ group presented a higher RPP_mean_ than CAC^−^, indicating more cardiac work for this group due to increased SBP. Since the blood pressure was measured after the exercise stop and not necessarily measured instantaneously the second the participant stopped, mean HR during the stop was used to calculate RPP_mean_. For participants who cycled the entire hill, RPP_max_ is also significant, and the *P*‐value decreases for both parameters, suggesting that increased external load yields larger discrepancies in cardiac work between the two groups.

### 4.1 HRV

The CAC^+^ group exhibited a lower low‐frequency component of HRV directly after the exertion. Previously, HRV_LF_ was thought to index cardiac sympathetic tone (Akselrod et al., 1981), but more recent research has shown that HRV_LF_ may reflect vagal activity and cardiovagal baroreflex modulation instead (Rahman et al., 2011; Reyes del Paso et al., 2013). It has been suggested that changes in HRV_LF_ are mediated through the baroreflexes’ influence on autonomic nervous system activity (Goldstein et al., 2011), with reduced cardiovagal baroreflex sensitivity shown to be associated with higher mean blood pressure (Zhou et al., 2023). Although HRV is usually measured during rest, a possible advantage of measuring directly after heavy physical exercise, is the exaggerated blood pressure response potentially accentuating HRV_LF_ by the mechanism mentioned earlier. This facilitates the use of HRV_LF_ as a non‐invasive parameter for assessing blood pressure regulation and the presence of calcified arterial plaques in otherwise asymptomatic individuals.

The logistic regression in Table 4 shows that HRV_LF_ predicts CAC^+^ independently of blood pressure. Hoshi et al. (2023) found that in participants aged 49 years or older, there is a significant association between HRV_LF_ and CAC regardless of hypertension and other clinical factors, suggesting autonomic neuropathy as a possible cause. Jae et al. (2017) reported that attenuated HR recovery after exercise was associated with advanced CAC, independent of other haemodynamic confounders. With HR recovery being a cardiovagal function marker, this supports the hypothesis that vagal function is affected in individuals with coronary arterial plaques. This study's 5 min HRV measurements were carried out after a heavy physical exertion during which sympathetic reduction and vagal reactivation occurs, possibly highlighting impaired autonomic function through HRV_LF_ in the CAC^+^ group. Thus, the lowered HRV_LF_ component for individuals with coronary artery calcification may be a combination of lowered cardiovagal baroreflex modulation and impaired autonomic function. Furthermore, Zambach et al. (2023) found no significant association between resting HR and CAC, but hypothesized a stronger correlation in more severe cases of cardiovascular dysfunction. The results in this study indicate that HRV measured directly after prolonged high‐intensity exercise may be more sensitive to CAC than resting HR, suggesting that HRV post‐exercise could be a valuable marker for early detection of CAC.

### 4.2 Strengths and limitations

This research is an observational study in which the study participants represent a selective cohort. Further, the Mann–Whitney test does not provide information about the magnitude of difference or effect size. However, it does not rely on specific assumptions about the underlying data distribution, making it robust and resilient against outliers, minimizing the consequences of measurement inaccuracies.

The analysis and interpretation of HRV data measured during exercise is challenging. HRV analysis relies significantly on the chosen artifact detection and correction methods. The data were corrected with several methods and thresholds, with all but one method yielding significant *P*‐values for the low‐frequency component. This method gave a significant *P*‐value when removing participants who stepped off the bike. Therefore, it is reasonable to conclude that the HRV_LF_ component is significantly lower for the CAC^+^ group than for the CAC^−^ group.

There is a significant age difference between the two groups, and although not statistically significant, there is a tendency towards higher resting SBP and lower ${\dot{V}}_{O_{2}\max}$ in the CAC^+^ group, as seen in Table 1. Thus, whether the differences between the two groups at THH are inherently linked to CAC or other shared associations should be assessed. Higher cardiorespiratory fitness is associated with lower blood pressure (Pepera et al., 2022) and improved autonomic function (Fu & Levine, 2013). Two logistic regressions on presence of CAC were carried out to account for all significant differences between the two groups in Table 2, as well as for established risk factors of atherosclerosis, such as age, sex, body mass index, maximum HR, ${\dot{V}}_{O_{2}\max}$, smoking, resting SBP and resting DBP. In both regressions, HRV_LF_ and the increase in DBP from baseline remained significant independent predictors of CAC. Thus, although we have not accounted for every potential confounding factor, the findings suggest that HRV_LF_ and the increase in DBP from baseline are independent predictors of CAC as far as our data indicate.

Finally, it should be noted that of the 56 participants included in this study, 12 stepped off their bikes and walked a portion of THH. These participants had significantly lower ${\dot{V}}_{O_{2}\max}$ compared to those who cycled the entire hill and included six participants from CAC^−^ and six from CAC^+^. Removing these participants further lowered the *P*‐value of all significant results, strengthening the results and underscoring the impact of high‐intensity exercise on the measured parameters.

### 4.3 Conclusion

The present study demonstrates significant alterations in haemodynamic measures and HRV_LF_ following prolonged high‐intensity endurance exercise in individuals with CAC. In multivariable analysis, HRV_LF_ remained an independent predictor of CAC despite adjusting for potential influencing factors, such as age, sex, smoking, resting blood pressure, maximal HR and ${\dot{V}}_{O_{2}\max}$. These findings indicate an adverse autonomic response to high‐intensity endurance exercise in individuals with CAC, suggesting a potential clinical role for HRV_LF_ in monitoring of individuals with CAC.

## AUTHOR CONTRIBUTIONS

Jakob Svane: Conceptualization; methodology; software; formal analysis; writing—original draft. Tomasz Wiktorski: Supervision. Trygve Eftestøl: Writing—review and editing. Stein Ørn: Conceptualization; methodology; formal analysis; supervision; writing—review and editing. All authors have read and approved the final version of this manuscript and agree to be accountable for all aspects of the work in ensuring that questions related to the accuracy or integrity of any part of the work are appropriately investigated and resolved. All persons designated as authors qualify for authorship, and all those who qualify for authorship are listed.

## CONFLICT OF INTEREST

The authors declare no conflicts of interests.

## FUNDING INFORMATION

This research received no external funding.

## Data Availability

The data that support the findings of this study are available from the corresponding author upon reasonable request. Software implementation of HRV data correction can be found at: https://github.com/jakobsv97/HRV‐CORRECTION‐ARIMA.
